# Identification of environmental aquatic bacteria by mass spectrometry supported by biochemical differentiation

**DOI:** 10.1371/journal.pone.0269423

**Published:** 2022-06-03

**Authors:** Natalija Topić Popović, Slavko Kepec, Snježana P. Kazazić, Ivančica Strunjak-Perović, Krunoslav Bojanić, Rozelindra Čož-Rakovac

**Affiliations:** 1 Laboratory for Aquaculture Biotechnology, Ruđer Bošković Institute, Zagreb, Croatia; 2 Scientific Centre of Excellence for Marine Bioprospecting-BioProCro, Ruđer Bošković Institute, Zagreb, Croatia; 3 Virkom d.o.o., Public Water Supply and Wastewater Services, Virovitica, Croatia; 4 Faculty of Medicine, Josip Juraj Strossmayer University of Osijek, Osijek, Croatia; 5 Laboratory for Mass Spectrometry, Ruđer Bošković Institute, Zagreb, Croatia; Fisheries and Oceans Canada, CANADA

## Abstract

In this study, the purposefulness of using the API20E biochemical identification system as a supportive tool for enhancing the discrimination of environmental bacteria by MALDI-TOF MS method was evaluated. The identification results of MALDI-TOF MS and API20E for 321 Gram-negative strains isolated from the riverine freshwater and its sediment, and from the tissues of fish from the same water body were compared. Of 190 isolates identified with probable to highly probable species-level identification, and secure genus to probable species identification, 14 isolates (7.37%) had identification score over 2.300, and from the same group 19 isolates (10%) had excellent or very good identification to the genus by API20E system. With regard to agreement at genus level, out of 231 strains with genus designation available by API20E at any level of identification reliability, MALDI-TOF MS genus identification agreed in 163 (70.6%) strains. Of these, 135 (82.8%) were *Aeromonas* species and the remaining isolates belonged to 7 different genera. Although API20E resulted in frequent misidentification due to a limited profile index, its individual biochemical reactions might contribute to overall characterization of isolates. For example, for all reliable *A*. *hydrophil*a strain identifications with MALDI-TOF MS, ONPG, GLU and OX reactions were unarguably positive for all fish and water/sediment isolates, whereas only fish isolates yielded additional 100% positive TDA and VP reactions. Thus, after initial identification with MALDI-TOF MS, environmental isolates with lower identification scores should be further analyzed. Before commencing confirmatory testing with nucleic acid-based methods, biochemical API20E tests could be applied as a purposeful and inexpensive identification support in targeting better identification accuracy. In this study, this was particularly evident with *A*. *hydrophila*, *Chryseobacterium* sp. and *Pseudomonas* sp. This identification strategy could significantly resolve methodological and cost-related shortcomings frequently occurring with large number of environmental isolates.

## Introduction

There is a vast microbial diversity on Earth, potentially 10^11^–10^12^ microbial species, but more than 99% of them remain undiscovered [1,2]. Only a small fraction of environmental bacteria can be cultured [3–6]. Methods, which give information on bacterial composition, their community structures, and their genetic diversity without cultivation, have been introduced. They include the real-time PCR, 16S ribosomal RNA (16S rRNA) sequencing, denaturing and temperature gradient gel electrophoresis (DGGE/TGGE), terminal restriction fragment length polymorphism (T-RFLP), fatty acid methyl esters (FAME) analysis, next generation sequencing (NGS), however, they remain time-consuming, costly and laborious when identifying a large number of bacterial isolates [1,7]. Accordingly, culture-dependent identification methods, requiring cultivation of cultivable bacteria, need to be very precise and reliable. Prompt and accurate identification of environmental bacteria is vital, particularly for disease-causing, zoonotic and opportunistic bacteria. The identification of environmental and particularly aquatic bacteria comes with challenges. They include discrimination between closely related environmental strains, rapid identification in some disease outbreaks, and identification of rare or less frequent microorganisms which are difficult to discriminate with classical techniques [8]. Most of them can be successfully resolved with the application of matrix-assisted laser desorption/ionization time of flight mass spectrometry (MALDI-TOF MS).

MALDI-TOF MS is a powerful tool for bacterial diagnostics, allowing identification in a few minutes [9]. It analyzes the proteome of treated bacterial cells and generates protein mass spectra which are used to group and identify bacteria. The protein mass spectra contain *m/z* peaks relating to ribosomal proteins relative to their high content in bacterial cells [10,11]. The unknown bacteria are identified at genus, species or sub-species taxonomic levels as the mass signals are compared with mass spectra from reference bacterial strains collected in a dedicated mass spectra library or with publicly available proteomics/genomics data [12,13]. In this work the database used for identification of tested bacteria was the Biotyper software (Bruker Daltonics, Bremen, Germany) which comprises over 5000 bacterial species, mostly of clinical significance. Nonetheless, in cases of not reliable identification or probable genus identification, often encountered with aquatic isolates, additional tests might be required to enhance the differentiation of a tested strain. To that end, biochemical tests which can identify bacteria to genus, species or biotype, can be employed [9,14]. Biochemical tests, namely API20E panels (bioMerieux, Marcy l’Etoile, France) are designed to give fast and efficient, standardized and economical identification of non-fastidious Gram-negative bacteria and Enterobacteriaceae based on extensive databases. Their identification rates are based on the likelihood of a match between the unknown bacterial profile and the database profile, the relative value between the likelihood of the first and the likelihood of the second choices, and the number of tests against the first choice [15].

In order to identify and characterize a large number of environmental isolates, and to determine which of them, if any, should be selected for further molecular discrimination procedures, we hypothesize that it is advisable to run both mass spectrometry as a first-line test and phenotypical/biochemical procedures as complementary tests. To test that premise, we compared the identification results of MALDI-TOF MS and API20E for 321 Gram-negative bacterial strains isolated from the riverine freshwater and its sediment, and from the tissues of fish from the same water body. We evaluated the purposefulness of using the biochemical identification system as a supportive tool for enhancing the discrimination of environmental bacterial isolates by MALDI-TOF MS method, taking into consideration the genus-level and species-level identification results.

## Materials and methods

### Bacterial strains

A total of 321 Gram-negative bacterial strains from our RBI laboratory collection were studied. They involved 110 strains from the tributaries of the river Drava (Croatia), 20 strains from its sediment, and 190 strains previously retrieved from the gills and internal tissues of fish from the same water body. The strains were defrosted from -86°C storage and streaked on a general-purpose medium (Tryptone Soya Agar, Oxoid Ltd., England, UK). The plates were incubated at 22°C. Fresh bacterial growth (24 h) was used for all analyses.

### Matrix-assisted laser desorption/ionization time-of-flight mass spectrometry (MALDI-TOF MS)

MALDI-TOF MS was performed with a bench-top Bruker Microflex LT mass spectrometer equipped with the Bruker Biotyper 3.0 software (Bruker Daltonik, Bremen, Germany) system. After being cultured at 22°C for 24 h or until the occurrence of colonial growth isolates were analyzed by the full extraction method (EtOH-FA) in triplicates, with a total of 963 measurements. The extraction method was performed as in Kazazić et al. [16]: a loopful of a bacterial colony from each strain was suspended in 300 μL of LC-MS-grade water (Fisher Chemical, St. Louis, MO) and immediately vortexed. Subsequently, 900 μL of 100% ethanol (Kemika, Croatia) was added to the suspension, vortexed and centrifuged at 16 000 *g* for 2 minutes. The supernatant was discarded and the pellet recentrifuged. After discarding the supernatant, the pellet was dried at RT and resuspended in 20 μL of 70% formic acid. The suspension was mixed by pipetting and 20 μL of acetonitrile was added, mixed and centrifuged at 16 000 *g* for 2 min. Before overlying with 1 μL (10 mg/mL) of MALDI matrix (α-Cyano-4-hydroxycinnamic acid, Bruker Daltonik), 1 μL of supernatant was added to each spot on a 96-spot polished stainless steel target plate and allowed to dry.

For validation of runs, the system was calibrated with a bacterial test standard *Escherichia coli* DH5 alpha spiked with two additional pure proteins (RNAse A and myoglobin) for mass ranges 4–17 kDa. Ions were captured in the positive linear mode with mass ranges between 2 and 20 kDa, and positive ions were extracted at accelerated voltage of 20 kV. Spectra with the sum of the respective ions were acquired by 240 laser shots in different regions of every target plate spot.

Mass spectra were matched to the reference mass spectra in the database. Bruker Biotyper 3.0 software (Bruker Daltonik) was used to analyze the spectra The software automatically classifies identification results according to their log score values. In order to minimize random effects, data obtained with replicate measurements were added to the calculation. By using the software interface for data interpretation and visualization of the results, the algorithms of the measured data were recorded as logarithmic scores between 0 and 3.0. The identification criteria were as follows: a calculated log score of 2.300 to 3.000 indicated highly probable species identification, a score of 2.000 to 2.299 indicated secure genus identification and probable species identification, a score of 1.700 to 1.999 indicated probable genus identification, while a score < 1.700 was considered unreliable. Therefore, strains with scores > = 2.0 were considered as identification at species level, > = 1.7 and < 2.0 as genus level, and < 1.7 as unreliable identification.

### API (Analytical Profile index) 20E system

The API20E system (bioMerieux, Marcy l’Etoile, France) comprises 23 standardized miniaturized biochemical tests in an inoculation strip with 20 microtubes, which contain dehydrated substrates. The bacterial inoculum was prepared with an overnight culture. The microtubes were inoculated with a bacterial suspension, which reconstituted the media. The API20E panels were used according to manufacturer’s instruction, with some alterations as described in Topić Popović et al. [17]. The incubation temperature for the strips was maintained at 22°C, the incubation time was 24–72 h, a suspension of 1.5% saline was used for the inoculum, while sterile mineral oil was used for sealing the cups for the fermentation of sugars. The biochemical tests investigated were the following: ß-galactosidase (ONPG), arginine dihydrolase (ADH), lysine decarboxylase (LDC), ornithine decarboxylase (ODC), citrate utilization (CIT), H2S production (H2S), urease (URE), tryptophane deaminase (TDA), indole production (IND), Voges–Proskauer (VP), gelatinase (GEL), glucose (GLU), mannitol (MAN), inositol (INO), sorbitol (SOR), rhamnose (RHA), saccharose (SAC), melibiose (MEL), amygdalin (AMY), arabinose (ARA), and cytochrome oxidase (OX). The reactions were read by using the APILAB software provided by the manufacturer. The obtained identification results for the readings included: excellent identification to the genus, very good identification to the genus, very good identification, good identification, acceptable identification, unacceptable profile, doubtful profile, low discrimination, and uninterpretable. For the purposes of this study, the API20E identifications were considered as species only if having excellent and very good levels of result reliability. They were considered as genus if having good and acceptable levels of reliability, and as unreliable for all other levels of result reliability. The biochemical data, along with the bionumerical profile result from each strip were used for a comparison with the MALDI-TOF MS identification score.

### Data analyses

All exploratory data analyses and visualizations were performed using R v4.1.2 (R: A language and environment for statistical computing. R Core Team (2021), URL: https://www.R-project.org/) in RStudio IDE v2021.09.0 (RStudio, PBC (2021), URL: https://www.rstudio.com/)).

## Results

**Fig 1:**
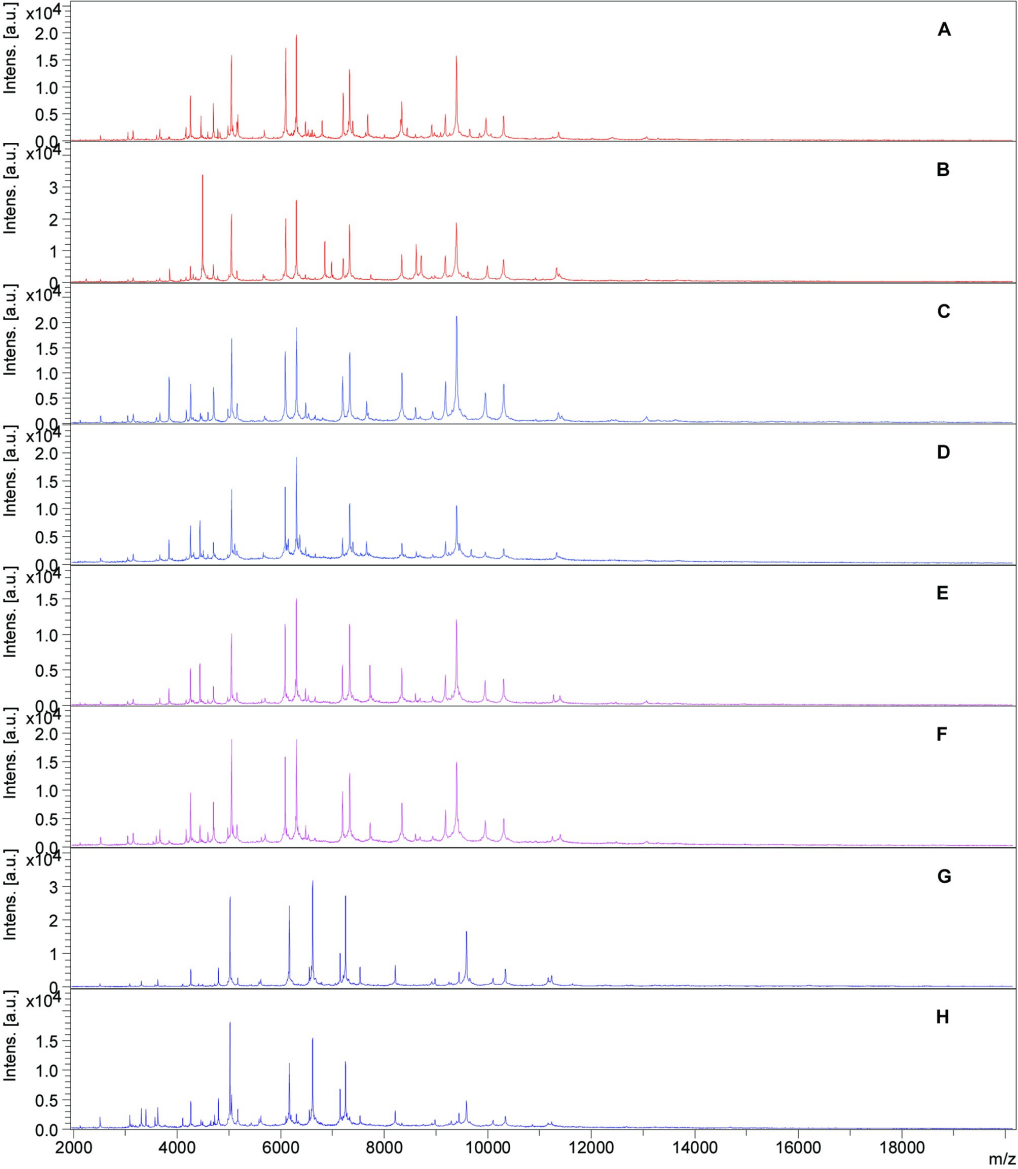
Representative MALDI-TOF mass spectra derived from analysis of bacterial isolates from the riverine freshwater (tributaries of the river Drava, Croatia) and fish from the respective water bodies. Mass spectra are as follows: *Aeromonas veronnii* A) from water, B) from fish; *A*. *popoffii* C) from water, D) from fish; *A*. *bestiarum* E) from water, F) from fish; *Shewanella baltica* G) from water, H) from fish.

Of 321 strains processed in parallel with MALDI-TOF MS and API20E, 44 strains were unidentifiable with both methods, *i*.*e*. having unreliable identification even at genus level. Of the remaining 277 strains for further exploration between the two methods, 113 were isolated from water/sediment and 164 from fish samples. MALDI-TOF MS identified 76 (67.3%) isolates with probable to highly probable species-level identification (and secure genus identification) and 37 (32.7%) isolates with probable genus identification in water/sediment, and 108 (65.9%) and 56 (34.1%) strains, respectively in fish samples. API20E identified 27 (23.9%) strains at genus level, 1 (0.9%) at species level, and 85 (75.2%) with unreliable identification in water/sediment and 12 (7.3%), 1 (0.6%), and 151 (92.1%) strains, respectively in fish samples. The full data with all levels of identification reliability by API20E is presented in Table 1 and complete raw data at strain level is presented in S1 Table. Overall, of 184 isolates identified with probable to highly probable species-level identification, and secure genus to probable species identification by MALDI-TOF MS, 15 isolates (8.2%) had identification score over 2.3, and from the same group 21 isolates (11.4%) had excellent or very good identification by API20E system. Representative mass spectra of selected bacterial species from fish and water/sediment samples are presented in Fig 1.

**Table 1:** Levels of reliability in results of bacterial identification between API20E and MALDI-TOF MS methods applied to 164 strains isolated from fish and 113 from water/sediment samples.

Method	Level	Fish	Water/Sediment	Total
MALDI-TOF MS	Genus	56	37	93
	Species	108	76	184
API20E	Excellent	4	1	5
	Very good	3	14	17
	Good	5	7	12
	Acceptable	1	6	7
	Doubtful	65	24	89
	Low discrimination	19	3	22
	Unacceptable	63	58	121
	Uninterpretable	3	0	3
	Not valid	1	0	1

**Fig 2:**
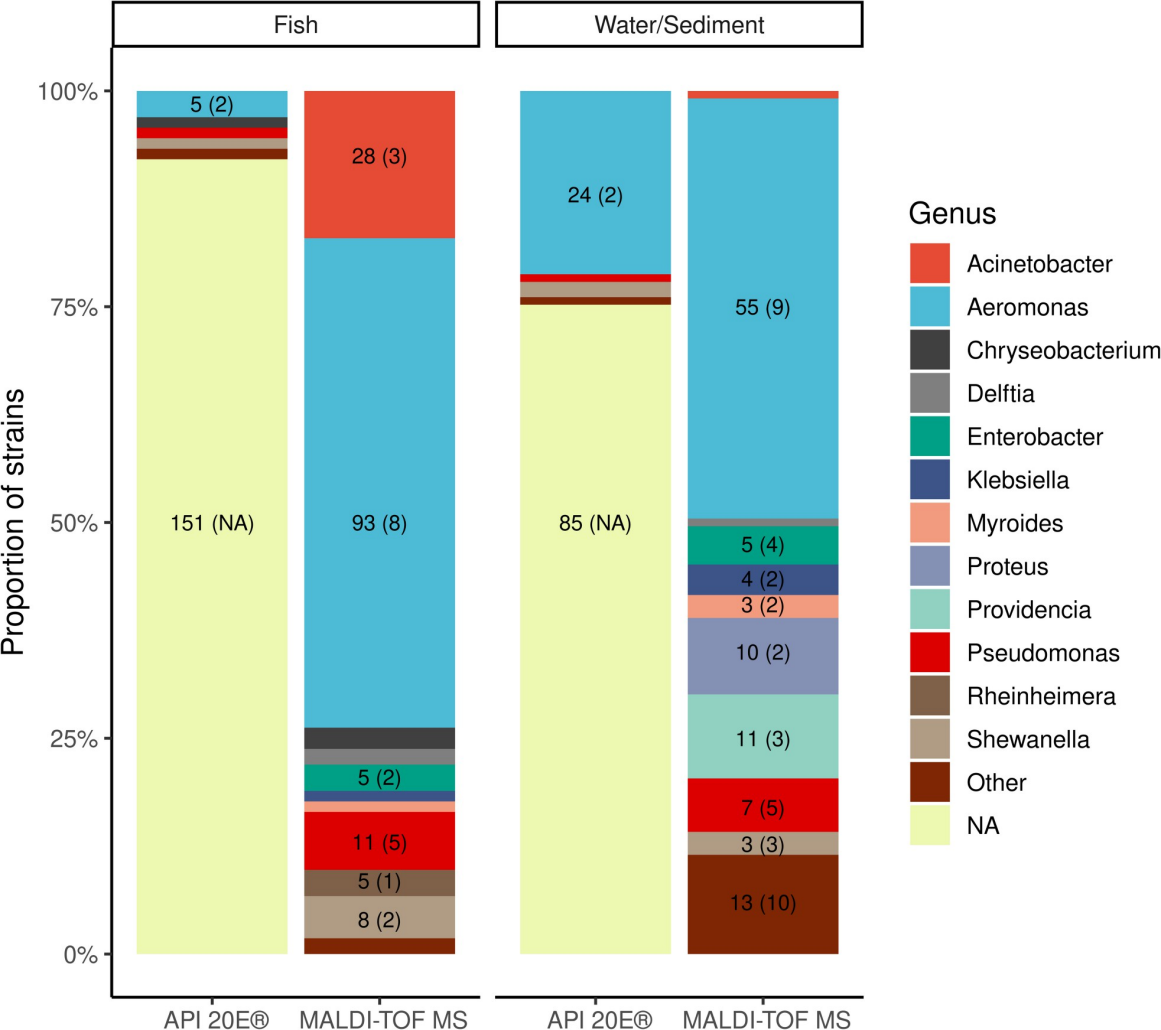
Dominant bacterial genera identified by MALDI-TOF MS and by API20E from water/sediment and from fish tissues. The numbers in columns refer to the total number of isolates in that particular genus; the numbers in brackets refer to the number of species of that particular genus. The *NA* category pertains to the API20E strips with unreliable identification at the genus level. The *Other* category is composed of genera with up to 2 strains.

With regard to species richness and distribution and accounting for the level of identification obtained, MALDI-TOF MS identified 39 species from 24 genera in total, of which there were 29 species from 20 genera in water/sediment and 21 and 13, respectively in fish samples. On the other hand, API20E identified 2 species and 6 genera in total, of which there was 1 species and 3 genera in water/sediment and 1 and 6, respectively in fish samples. Unreliable identification of API20E was at 75% (85/113) in water/sediment samples which was better than 92% (151/164) observed in fish samples but the difference was not significant (p = 0.3). Overall, the vast majority of all identified strains by MALDI-TOF MS belonged to genera *Aeromonas* (53.4%), followed by *Acinetobacter* (10.5%), *Pseudomonas* (6.5%), *Providencia* (4.0%), *Shewanella* (4.0%), *Enterobacter* (3.6%), *Proteus* (3.6%) and a long list of other genera represented by fewer strains (S1 Table). However, the ratio of identified species did not correspond between water/sediment and fish tissue isolates and is shown in Fig 2. That is, with regard to agreement at genus level, out of 231 strains that had genus designation available by API20E at any level of identification reliability, MALDI-TOF MS genus identification agreed in 163 (70.6%) strains. Important to note is that of these 163 strains, 135 (82.8%) were *Aeromonas* species and the remaining isolates belonged to 7 different genera. Using a subset of API20E results with reliable identification at genus level, the concordance with MALDI-TOF MS genus identification remained high as 35 strains out of 41 (85.4%) available had the same genus identified, yet again 26 (63.4%) of these strains belonged to *Aeromonas* genus. With regard to reliable identification at species level, API20E identified only 2 strains, one *Serratia liquefaciens* from water/sediment, and one *Shewanella putrefaciens* from fish sample. MALDI-TOF MS identification agreed with *S*. *liquefaciens* strain at the species level yet the *S*. *putrefaciens* strain was identified as *Delftia acidovorans* with a reliable genus identification. The main reason for a high genus and low species agreement between the two methods lies within aeromonads strains that API20E at species level attributed to either *A*. *hydrophila* group 1 or *A*. *hydrophila* group 2. These two groups are composed of several species and further tests are required to differentiate them yet their identification results were derived from 55 (7–9 digit) identification profiles ranging from excellent identification to the genus down to low discrimination. Concordance in genus identification between the two methods for all strains is presented in S2 Table.

Since reliable taxonomic identification at both the species and genus level was relatively low with API20E compared to MALDI-TOF MS, the biochemical profiles obtained were further explored according to MALDI-TOF MS results. The observed phenotypic variation within single species identified by MALDI-TOF MS was quite high as presented in Table 2. For instance, out of 22 species that had more than one strain available, only 8 (36.4%) species returned a single biochemical profile, On one end, *A*. *hydrophila* was shown to have the highest phenotypic variation of all species as 14 strains from water/sediment returned 13 profiles and 5 strains from fish returned 4 biochemical profiles. On the other end, consistent biochemical profiles were relatively rarely observed and *Shewanella baltica* had the highest consistency with 5 strains from fish showing identical profiles. Interestingly, *Acinetobacter johnsonii* was the only species well identified with MALDI-TOF MS, but unidentifiable due to no reactions in the API strip, for three isolates (12.5% of *A*. *johnsonii* isolates).

**Table 2:** Variation in API20E phenotypic profiles* (expressed as % of positive results) within bacterial species as confirmed by MALDI-TOF MS. Isolates were sourced from fish and water/sediment (W/S) and taxa represented by only one strain were not included.

MALDI Genus and species		Sample	Strains (profiles)	ONPG	ADH	LDC	ODC	CIT	H2S	URE	TDA	IND	VP	GEL	GLU	MAN	INO	SOR	RHA	SAC	MEL	AMY	ARA	OX
*Acinetobacter johnsonii*		Fish	13 (9)	15.4	23.1	23.1	15.4	15.4	7.7	0	23.1	23.1	53.8	15.4	15.4	15.4	7.7	7.7	7.7	15.4	7.7	7.7	7.7	30.8
*Aeromonas*	*bestiarum*	Fish	4 (2)	100	100	100	0	75	0	0	25	100	100	100	100	100	0	0	25	100	0	100	100	100
		W/S	2 (2)	100	100	100	50	50	0	0	50	50	50	100	100	100	0	0	100	100	0	50	100	100
	*encheleia*	Fish	2 (1)	100	100	100	0	0	0	0	100	100	0	0	100	100	0	0	0	100	0	100	100	100
	*hydrophila*	Fish	5 (4)	100	80	80	0	0	0	0	100	80	100	40	100	80	20	0	0	80	0	60	60	100
		W/S	14 (13)	100	92.9	85.7	21.4	71.4	14.3	7.1	64.3	85.7	57.1	100	100	92.9	14.3	42.9	35.7	92.9	21.4	92.9	92.9	100
	*ichthiosmia*	Fish	2 (1)	100	100	100	0	100	0	0	100	100	100	100	100	100	0	0	0	100	0	0	0	100
		W/S	2 (2)	100	100	100	0	50	50	0	100	100	0	0	100	100	0	0	0	100	100	50	50	100
	*media*	Fish	5 (3)	100	100	40	40	40	0	0	100	100	40	100	100	100	0	40	0	100	0	100	60	100
		W/S	3 (3)	100	66.7	0	0	0	0	0	66.7	66.7	66.7	66.7	100	100	0	0	0	100	0	66.7	100	100
	*popoffii*	Fish	3 (3)	100	100	66.7	0	33.3	0	0	33.3	100	33.3	66.7	100	100	0	0	0	66.7	0	66.7	33.3	100
		W/S	5 (4)	100	100	80	0	0	20	0	0	40	100	100	100	100	0	80	20	100	0	100	100	100
	*sobria*	Fish	7 (4)	100	100	100	14.3	100	0	0	100	57.1	71.4	57.1	100	100	14.3	14.3	0	100	0	100	28.6	100
	*veronii*	Fish	44 (29)	100	95.5	97.7	9.1	68.2	0	0	100	95.5	65.9	54.5	100	100	6.8	9.1	6.8	97.7	11.4	50	27.3	95.5
		W/S	15 (10)	93.3	93.3	86.7	13.3	100	6.7	6.7	73.3	73.3	80	100	100	100	6.7	6.7	13.3	100	26.7	53.3	26.7	100
*Enterobacter cloacae*		Fish	3 (3)	100	100	0	100	100	0	0	33.3	0	100	33.3	100	100	66.7	100	100	100	100	100	100	100
*Escherichia coli*		W/S	2 (2)	100	50	100	50	50	0	0	0	50	50	0	100	100	0	100	100	100	50	50	100	50
*Klebsiella*	*oxytoca*	Fish	2 (1)	100	0	100	0	100	0	100	100	100	100	0	100	100	100	100	100	100	100	100	100	100
	*pneumoniae*	W/S	3 (3)	100	33.3	100	0	100	0	33.3	100	33.3	100	33.3	100	100	100	100	100	100	100	100	100	33.3
*Myroides odoratimimus*		W/S	2 (1)	100	100	0	100	100	100	100	100	0	0	100	100	0	0	0	0	100	0	100	0	100
*Proteus vulgaris*		W/S	4 (3)	100	50	50	50	75	100	100	100	100	25	100	100	100	25	75	100	100	75	75	100	75
*Providencia*	*alcalifaciens*	W/S	2 (1)	100	100	0	100	100	100	100	100	100	100	0	100	100	0	100	100	100	100	100	100	100
	*rettgeri*	W/S	4 (3)	100	50	75	0	100	50	100	100	50	50	75	100	100	100	100	75	100	75	100	75	75
*Pseudomonas*	*chlororaphis*	Fish	2 (1)	0	100	0	0	100	100	0	100	0	0	100	100	0	0	0	0	0	100	0	0	100
	*fragi*	Fish	3 (2)	0	100	0	0	100	0	0	33.3	0	0	0	100	0	0	0	0	0	100	0	100	100
	*putida*	W/S	2 (2)	50	50	50	50	100	50	50	100	50	0	50	50	50	0	50	50	50	50	50	50	100
*Rheinheimera soli*		Fish	2 (1)	0	0	0	0	0	0	0	100	0	0	0	0	0	0	0	0	0	0	0	0	0
*Shewanella baltica*		Fish	5 (1)	100	100	100	0	0	100	0	100	100	100	100	0	100	0	100	0	100	0	100	0	100

* ß-galactosidase (ONPG), arginine dihydrolase (ADH), lysine decarboxylase (LDC), ornithine decarboxylase (ODC), citrate utilization (CIT), H2S production (H2S), urease (URE), tryptophane deaminase (TDA), indole production (IND), Voges–Proskauer (VP), gelatinase (GEL), glucose (GLU), mannitol (MAN), inositol (INO), sorbitol (SOR), rhamnose (RHA), saccharose (SAC), melibiose (MEL), amygdalin (AMY), arabinose (ARA), and cytochrome oxidase (OX).

## Discussion

In this study we compared the identification results of MALDI-TOF MS and API20E for 321 Gram-negative bacterial strains isolated from the riverine freshwater and its sediment, and from the tissues of fish from the same water body. We aimed to evaluate the purposefulness of using the API20E biochemical identification system as a supportive tool for enhancing the discrimination of environmental bacterial isolates by MALDI-TOF MS.

It is indisputable that MALDI-TOF MS outperforms the biochemical test identification of various environmental bacteria, and just one of the many arguments is its taxonomic database, constantly upgraded with additional mass spectra. API20E yields frequent misidentification and has a limited profile index [18], but when observing its individual biochemical reactions, it is clear that they might contribute to overall characterization of an isolate. Although API20E strips failed to identify a sizable number of isolates, some biochemical traits were typical for certain strains in this study. For example, for all *A*. *hydrophil*a isolates which were confirmed by both systems to the genus, ONPG, ADH and LDC reactions were positive in 85.71% of strains, while AMY, ARA and OX were positive in 92.86% of strains. Besides, in their API20E excellent identification result, positive were also CIT, IND, VP, GEL, GLU, MAN and SAC biochemical tests, which can be attributed to its genus, since in several cases they did not correspond to the strain identification with MALDI-TOF MS. However, for all reliable *A*. *hydrophil*a strain identifications with MALDI-TOF MS, ONPG, GLU and OX were unarguably positive for all fish and water/sediment isolates, whereas only fish isolates yielded additional 100% positive TDA and VP reactions. This difference in *A*. *hydrophila* biochemical profile might be related to their plasmid diversity as plasmids contain genetic determinants responsible for their replication and stability, but they may also carry genes that help bacteria adapt to different environments [19].

For pseudomonads, good and very good identification to the genus always had positive CIT and OX and negative INO and VP biochemical reactions. They corresponded with the MALDI-TOF MS identification to the genus in all cases. Therefore, in instances of the MALDI-TOF MS probable or unreliable genus identification, this biochemical trait might prove useful in confirmation of the genus. We noticed similar with *Chryseobacterium*, identified by MALDI-TOF MS as *C*. *scopthalmum* with probable genus identification score, but identified as *C*. *meningosepticum* with good to the genus identification result by API20E, suggesting that positive IND and GEL biochemical reactions might be supportive for the identification of the genus. Comparably, positive H_2_S and OX reactions might be supportive tests for the confirmatory genus identification of *Shewanella* when MALDI-TOF MS indicates only to the probable or unreliable genus identification. Indeed, for environmental bacteria many databases are incomplete or contain reference mass spectra obtained from clinical and reference collection strains under specific and stringent culture conditions, not necessarily reflecting the strain (biochemical) requirements in their natural environment [20].

Some organisms, such as *Escherichia coli* and *Shigella* species have nearly identical genetic profiles and protein fingerprints and are therefore not easily distinguished [21]. Until recently, MALDI-TOF MS was not able to discriminate between *E*. *coli* and *Shigella* species. A rapid classification method for the *E*. *coli-Shigella* phylogroup based on MALDI-TOF MS supported by MLVA genetic analysis was developed [22]. In this study, MALDI-TOF MS identified two isolates with secure genus and probable species identification as *E*. *coli*, which was not corroborated with a typical API20E profile. The two suggested API profiles only had positive MAN and SOR tests in common for supplemental characterization of *E*. *coli*.

Conversely, highly probable species identification (log score 2.300 and above) obtained by MALDI-TOF MS for *A*. *hydrophila*, *Citrobacter braakii*, *Enterobacter cloacae*, *P*. *putida* and *Serratia liquefaciens* having identical species identification with API20E ranging from doubtful to very good, might give weight to their bionumerical profile results. In particular, profile results for *A*. *hydrophila* 7446137 (doubtful), 7006127 (good), 7065124 (unacceptable), *C*. *braakii* 0324553 (doubtful), *E*. *cloacae* 3324573 (doubtful), *P*. *putida* 2220004 (doubtful) and *S*. *liquefaciens* 5307763 (very good to the genus) might thus be regarded as very good identification results. Reliable, highly probable species identification in a case of *A*. *veronii*, which API20E identified as possible *Vibrio fluvialis*, or its secure genus identification and probable species identification of three species which API20E identified as possible *V*. *cholera*, is not surprising. It was shown that motile aeromonads, occurring widely in water and sewage, might be misinterpreted as vibrios by API20E [23,24]. Their biochemical characteristics are variable and often result in misidentifications at the species and even genus level, with *Vibrio* being the most common misidentification. Some motile aeromonads give false positive or negative reactions for LDC, VP, GEL and fermentation of some sugars such as ARA, SOR and RHA [25]. Indeed, Israil et al. [26] demonstrated that API20E had limits in the diagnostics of the aquatic *Vibrio* and *Aeromonas* species, mainly being discordant for GEL, LDC, ADH, SAC, MAN and INO reactions. However, as more environmental bacterial profiles are added to the API20E database, the ability to corroborate identification results with mass spectrometry will surely increase and its identification value will enhance [15].

A cost of analysis of a large number of samples is certainly a limiting factor when making decisions on the identification strategy. In that sense, MALDI-TOF MS, although high-priced in initial acquisition, is inexpensive with low marginal costs, has a low sample volume requirement and readily interpretable data [14]. It reduced the time required for identification by 169-fold and the cost by 96-fold compared with gene sequencing in the work of Seng et al. [27]. It has a low cost of reagents, ease of performance and rapid turnaround time [28]. Contrarily, the high cost and the specialized training for the 16S rRNA gene sequencing makes it impossible to use in multiplex analyses [29]. The API system, on the other hand, is a well-established culture-based platform, easy to perform, requiring limited training, but in terms of cost per isolate cannot compete with MALDI-TOF MS [8].

In summary, after initial identification with MALDI-TOF MS, environmental isolates with lower identification scores should be further analyzed. Before commencing confirmatory testing with nucleic acid-based methods, such as 16S rRNA, their cost, labor involvement and long turnaround time should be taken into consideration [14]. In that sense, biochemical API20E tests, needing a few minutes to inoculate and ten minutes to interpret, are a purposeful and inexpensive substitute for conventional tube-based identification tests for Gram-negative bacteria when identification support is required in targeting better identification accuracy [21]. In this study, this was particularly evident with *A*. *hydrophila*, *Chryseobacterium* sp. and *Pseudomonas* sp. with their typical biochemical reactions to the genus level, although yielding insecure identification result. In further need of resolving ambiguities, confirmatory molecular identification of a small number of isolates could then be applied. This identification strategy could significantly resolve methodological and cost-related shortcomings frequently occurring with identification of a large number of environmental isolates. Nevertheless, the results of this study should be considered in the context of the strains evaluated as they derived from the distinct aquatic environment and tissues of fish originating from that respective habitat.

## Supporting information

S1 Table MALDI-TOF MS and API20E results for 277 bacterial strains isolated from riverine freshwater or sediment (113) and fish samples (164). Identification reliability for MALDI-TOF MS was used as provided by the manufacturer (log score 2.300 to 3.000—highly probable species identification, log score 2.000 to 2.299—secure genus identification and probable species identification, log score 1.7 to 1.999 –probable genus identification). All MALDI-TOF MS measurements were conducted in triplicates with EtOH-FA extraction method. API20E identification reliability levels were used as provided by the manufacturer. Data on isolated strains that were unidentifiable by both methods (44) are not included.
(XLSX)

S2 Table Concordance in identification reliability between MALDI-TOF MS and API20E methods. Taxa and levels of identification are sorted by MALDI-level identification result (reliable genus or species identification) and presenting the API-level of identification (reliable or unreliable identification).
(XLSX)
